# Fenofibrate reduces glucose-induced barrier dysfunction in feline enteroids

**DOI:** 10.1038/s41598-023-49874-9

**Published:** 2023-12-18

**Authors:** Charles K. Crawford, Aeelin Beltran, Diego Castillo, Muhammad S. Matloob, Mimoli E. Uehara, Mary L. Quilici, Veronica Lopez Cervantes, Amir Kol

**Affiliations:** Department of Pathology, Microbiology, and Immunology, School of Veterinary Medicine, University of California, Davis, CA USA

**Keywords:** Gastroenterology, Intestinal stem cells, Diabetes

## Abstract

Diabetes mellitus (DM) is a common chronic metabolic disease in humans and household cats that is characterized by persistent hyperglycemia. DM is associated with dysfunction of the intestinal barrier. This barrier is comprised of an epithelial monolayer that contains a network of tight junctions that adjoin cells and regulate paracellular movement of water and solutes. The mechanisms driving DM-associated barrier dysfunction are multifaceted, and the direct effects of hyperglycemia on the epithelium are poorly understood. Preliminary data suggest that fenofibrate, An FDA-approved peroxisome proliferator-activated receptor-alpha (PPARα) agonist drug attenuates intestinal barrier dysfunction in dogs with experimentally-induced DM. We investigated the effects of hyperglycemia-like conditions and fenofibrate treatment on epithelial barrier function using feline intestinal organoids. We hypothesized that glucose treatment directly increases barrier permeability and alters tight junction morphology, and that fenofibrate administration can ameliorate these deleterious effects. We show that hyperglycemia-like conditions directly increase intestinal epithelial permeability, which is mitigated by fenofibrate. Moreover, increased permeability is caused by disruption of tight junctions, as evident by increased junctional tortuosity. Finally, we found that increased junctional tortuosity and barrier permeability in hyperglycemic conditions were associated with increased protein kinase C-α (PKCα) activity, and that fenofibrate treatment restored PKCα activity to baseline levels. We conclude that hyperglycemia directly induces barrier dysfunction by disrupting tight junction structure, a process that is mitigated by fenofibrate. We further propose that counteracting modulation of PKCα activation by increased intracellular glucose levels and fenofibrate is a key candidate regulatory pathway of tight junction structure and epithelial permeability.

## Introduction

Diabetes mellitus (DM) is a metabolic disease that results in impaired glucose homeostasis. DM in humans has been coined a ‘new epidemic’ and is considered one of the world’s highest priority health problems^1^. DM is also a common naturally occurring endocrine disorder in household cats (*Felis catus)* that affects up to 0.7% of cats in the United States, and incidence rates of 2% occur in highly susceptible breeds such as Burmese cats^2,3^. Diabetic cats can provide valuable insight into human DM in addition to veterinary care, as cats are one of few species that experiences spontaneously occurring DM that closely resembles type 2 DM in humans with glucotoxicity-induced hyperinsulinemia, obesity-induced insulin resistance, and accumulation of pancreatic islet amyloid polypeptide^4^. As such, improved understanding of DM in cats holds the potential to improve veterinary healthcare outcomes while simultaneously providing highly translatable insight into human DM given the shared complex pathophysiology, chronic disease manifestation, and long-term medical care diabetic people and cats share^5^.

DM in cats and people is associated with multiple gastrointestinal co-morbidities collectively termed ‘diabetic enteropathy’^6,7^. The pathogenesis of diabetic enteropathy is complex and multifactorial and is thought to be driven by increased oxidative stress, neuroinflammation, reduced levels of nerve growth factors, structural vascular changes, and intestinal barrier dysfunction^6,8^. The intestinal epithelium forms a selective barrier that absorbs nutrients, water, and other important molecules while simultaneously blocking the entry of pathogens and harmful substances^9^. This barrier is comprised of a mucus layer over a monolayer of epithelial cells; the lamina propria, which contains immune cells, lies basal to the epithelial monolayer^10^. The epithelial cells are adjoined by a series of protein complexes: tight junctions, adherens junctions, and desmosomes^11^. Tight junctions are comprised of transmembrane and intracellular proteins found in the apical portion of intestinal epithelial cells, and they regulate paracellular transport across the intestinal epithelium^12–15^. DM is highly associated with increased permeability of the intestinal epithelium^6,16,17^ that alters paracellular transport and increases the risk of pathogenic entry and chronic diarrhea^18–20^. However, the precise direct role of hyperglycemia in this process is not well understood.

Recently, intestinal barrier dysfunction has been shown to be influenced by hyperglycemia in rodent models^8,21^. A high carbohydrate diet that induced intestinal barrier dysfunction in mice was associated with a reduction in expression of tight junctional proteins ZO-1 and Occludin, but this was in conjunction with increased inflammation, making it difficult to parse the direct effects of glucose versus inflammation on epithelial tight junctions^22^. In a human intestinal epithelial cancer cell line (CACO-2), apical (i.e. luminal) exposure to a high concentration of glucose altered tight junctional morphology as shown by increased tortuosity^8,23^. The independent effects of hyperglycemia on intestinal epithelium remain to be elucidated, especially in the context of feline DM.

Fenofibrate is an FDA-approved ligand of the peroxisome proliferator-activated receptor-alpha (PPARα) pathway that is used to treat patients with hyperlipidemia and dyslipidemia^24^. While the effects of fenofibrate on diabetic retinopathy, neuropathy, and atherosclerosis were investigated, its effect on diabetic enteropathy is poorly studied^25–28^. PPARα is a ligand-activated nuclear receptor transcription factor highly expressed in the small intestine that regulates numerous gene targets, especially related to metabolism, but it can also regulate ion channel function via non-transcriptional pathways^29–32^. PPARα activation protected against induced colitis in a rodent model for inflammatory bowel disease, and it enhanced intestinal barrier function in rhesus macaques with chronic gut inflammation and dogs with experimentally-induced DM^23,33,34^. In CACO-2 cells, treatment with fenofibrate improved barrier function, reduced junctional tortuosity, and increased the expression of Claudin-1, a tight junctional protein, following high glucose or inflammatory cytokine exposure^23^. While PPARα activation appears to have intestinal barrier protective properties, the specific mechanisms by which it may do so in DM are not fully understood. The influence of fenofibrate on Claudin-1 expression in dogs with experimentally-induced diabetes and tight junctional morphology in CACO-2 cells highlights tight junctions as a potential target^23,34^. PPARα activation, however, reduces the concentration of the inflammatory cytokines IL-8 and TNF-α, reduces expression of apical and basolateral glucose transport proteins, and protects against oxidative stress^23,35^. Thus, PPARα activation likely induces a complex response which attenuates intestinal barrier dysfunction. Its direct impact on the effects of hyperglycemia on feline intestinal epithelial permeability and resultant mechanisms are unknown.

We hypothesized that hyperglycemia directly induces intestinal barrier dysfunction in diabetic cats by disrupting intestinal epithelial tight junctions. Furthermore, we hypothesized that this can be reduced by fenofibrate. To investigate this, we utilized intestinal organoids (enteroids) derived from household cat intestinal tissue, providing a relevant in vitro model which recapitulates native intestinal epithelium^36^. We found that basolateral exposure of feline enteroids to high glucose concentrations, a model system for hyperglycemia, directly increases intestinal epithelial permeability and tight junction tortuosity, and that this effect is reduced when treated with fenofibrate. Surprisingly, tight junction gene expression was not altered by fenofibrate treatment. Rather, our data show that hyperglycemia increases PKCα activation, which is reduced by fenofibrate. Taken together, our data show that hyperglycemia induces intestinal barrier dysfunction in cat intestinal epithelium and highlights fenofibrate as a potential therapeutic for feline DM-induced intestinal barrier dysfunction.

## Materials and methods

### Enteroid generation

All experimental protocols were approved by the Institutional Animal Care and Use Committee at UC Davis. Feline intestinal tissue was obtained from intestinal resection and anastomosis surgery of the ileum due to an intestinal foreign body at the UC Davis William R. Pritchard Veterinary Medical Teaching Hospital. Intestinal tissue was healthy with no observable deviations. All tissue collection procedures were carried out in accordance with the American Veterinary Medical Association guideline, and informed consent was obtained from the cat owner. Discarded intestinal tissue was used for enteroid generation. Feline enteroids from one cat were generated (Supplementary Fig. S1) as previously described^37^ with minor protocol modifications in that a different growth medium was utilized based on separate previously published work^38^. Wells were provided 720μL of growth media containing 50% L-WRN conditioned media^39,40^, 50% DMEM/F12 media, 100U/mL penicillin/streptomycin, 25 µg/mL gentamicin (only for primary culture) supplemented with 10 μM Y-27632 (Tocris Bioscience), 10 mM Nicotinamide (Sigma), 1 mM N-acetylcysteine (Sigma), 10 nM Leu-Gastrin (Sigma), 1 × B27-insulin (Thermo), 500 nM LY2157299 (Thermo Fisher), 500 nM SB202190 (Sigma), 50 ng/mL mouse recombinant EGF (Thermo), and 3 μM CHIR99021 (only for primary culture or post-passage) (Tocris Bioscience). Media was changed every 3 days and organoids were passaged mechanically using a 25-gauge needle every 7–10 days.

### Treatment

Four days following passage, enteroids were provided with their normal growth medium containing a glucose concentration of 9.1 mM, or provided with growth medium containing added glucose (glucose concentrations of 22 mM or 44 mM above baseline with final concentrations of 31.1 mM and 53.1 mM) with or without fenofibrate (10 μM) (Sigma) or GLUT2-selective inhibitor (1 μM) (Life Chemicals #F0575-0046)^41^. Additionally, to ensure the effects of glucose treatment were not solely due to osmotic changes, some enteroids were provided growth medium containing the inert sugar, mannitol (22 mM, 44 mM) (Sigma Aldrich). Enteroids were treated for 24 h before FITC imaging, fixation, RNA extraction, or Western blot.

### FITC dextran permeability assay

Enteroids cultured in eight-well chamber slides (Thermo Scientific) were provided growth media containing 5ug/mL 4Kda fluorescein isothiocyanate-dextran (FITC) (Sigma Aldrich) for 1 h, then washed in PBS and imaged with a fluorescent microscope utilizing a 10 × objective (EVOS M5000, Thermo Fisher). FITC fluorescent intensity inside of the enteroids (average of three data points per enteroid) was normalized to FITC intensity outside of the enteroids (average of three data points per enteroid) via image analysis software (ImageJ) as previously described^37^.

### Immunofluorescence (IF) staining

Enteroids were cultured in eight-well chamber slides for immunocytochemical staining. Media was removed and organoids were fixed in 4% paraformaldehyde for 20 min at room temperature. Afterward, organoids were permeabilized in PBS containing 0.5% Triton X-100 (Sigma-Aldrich) for 20 min at room temperature. Wells were washed in IF buffer: PBS containing 0.2% Triton X-10 and 0.05% Tween (Fisher Scientific). They were then blocked in IF buffer containing 1% bovine serum albumin (Fisher Scientific) for 30 min at room temperature. After blocking, organoids were incubated in IF buffer containing 1% bovine serum albumin with primary antibodies overnight at 4 °C. Primary antibodies included ZO-1 (1A12, Invitrogen #33–9100), Cleaved Caspase-3 (Asp175, Cell Signaling #9661), and Ki67 (SP6, Invitrogen #MA5-14,520). Organoids were then washed in IF buffer, then incubated in IF buffer containing 1% bovine serum albumin with secondary antibodies for 1 h at room temperature. Organoids were washed in IF buffer, then incubated in IF buffer containing 0.1 µg/mL DAPI (Thermo Fisher) for 10 min at room temperature. The gasket of the chamber slide was then removed, one drop of ProLong Gold antifade reagent (Invitrogen) was placed into each well, and the slide was covered with a coverslip and allowed to cure for 24 h before being placed in darkness at 4 °C until imaging.

### Confocal microscopy and image analysis

After immunofluorescent staining for Ki67, Cleaved Caspase-3, or the tight junction protein, ZO-1, images were acquired on a TCS SP8 STED3x confocal microscope (Leica Microsystems) utilizing a 40x/1.3 oil immersion objective for Cleaved Caspase-3 (zoom 1.25) and a 63x/1.40 oil immersion objective for Ki67 (zoom 1.25) and ZO1 (zoom 3.25). ZO-1 images were processed via morphological segmentation plug-in (ImageJ) for tortuosity calculation, also known as the zigzag index^23,37,42,43^. The measured tortuosity was the ratio of segment length and Euclidian distance between two defined ZO-1 segment points^23^.

### RNA isolation and RT-quantitative PCR

Enteroids were collected and Matrigel was dissolved in ice-cold PBS. An RNeasy Plus Micro Kit (Qiagen) was used to extract total RNA from the enteroids, and spectrophotometry (Nanodrop, Thermo Fisher Scientific) was used to determine RNA concentration. A DNA-free DNase Treatment kit (Thermo Fisher) was used to remove gDNA and a First-strand cDNA Synthesis kit (OriGene) was used to synthesize cDNA. A StepOnePlus Real-Time PCR System (Applied Biosystems) with PowerUp SYBR Green Master Mix (Applied Biosystems) was used for PCR amplification. *Felis catus-*specific primers were designed with Primer Blast (NCBI). Primers were validated with melting curve analysis and amplicon size confirmation via gel electrophoresis. StepOne Software v2 1 was utilized for qPCR analysis with *Felis catus* GAPDH used as an endogenous control. Primers’ sequences can be found in Supplementary Table S1.

### Western blot

Enteroids were collected by pipette in ice cold PBS. Matrigel was dissolved in cold PBS and removed via centrifugation and supernatant removal. Enteroid cells were lysed and protein extracted with lysis buffer (150 mM NaCl, 50 mM Tris base, 1% NP-40, 0.25% deoxycholic acid, 0.1% SDS) with 1% Prometheus General Protease Inhibitor Cocktail (Genesee Scientific). Protein was quantified via BCA Protein Assay kit (Thermo Fisher). 20 µg of protein were loaded per well into a polyacrylamide gel (GenScript), separated by gel electrophoresis, then transferred onto a PVDF membrane (Thermo Scientific). Protein was probed with polyclonal antibodies against PKCα (Cell Signaling Technology #2056) or phosphor-(Ser) PKC substrate (Cell Signaling Technology #2261). Membranes were stripped and reprobed with an anti-GAPDH monoclonal antibody (Abcam #G041) as a loading control. Primary antibodies were diluted 1:1000. Prometheus ProSignal Femto chemiluminescent substrate (Genesee Scientific) was applied to membranes which were imaged with a KwikQuant Pro Imager (Kindle Biosciences). Densitometry analysis of bands was performed using ImageJ image analysis software.

### Statistical analysis

Experiments are separated by color and experimental data are presented as means ± SD. Data were examined for normality via Shapiro–Wilk test (α = 0.05). Comparisons of normal data were analyzed via one-way ANOVA (α = 0.05) with Holm-Šídák post hoc analysis. Comparisons of data not normally distributed were analyzed via Kruskal–Wallis non-parametric ANOVA (α = 0.05) with Dunn’s post hoc analysis. Statistical significance denotation: *p ≤ 0.05 | **p ≤ 0.01 | ***p ≤ 0.001 | ****p ≤ 0.0001.

## Results

### Hyperglycemia-like conditions increase epithelial permeability in feline enteroids, which is prevented by fenofibrate

**Figure 1:**
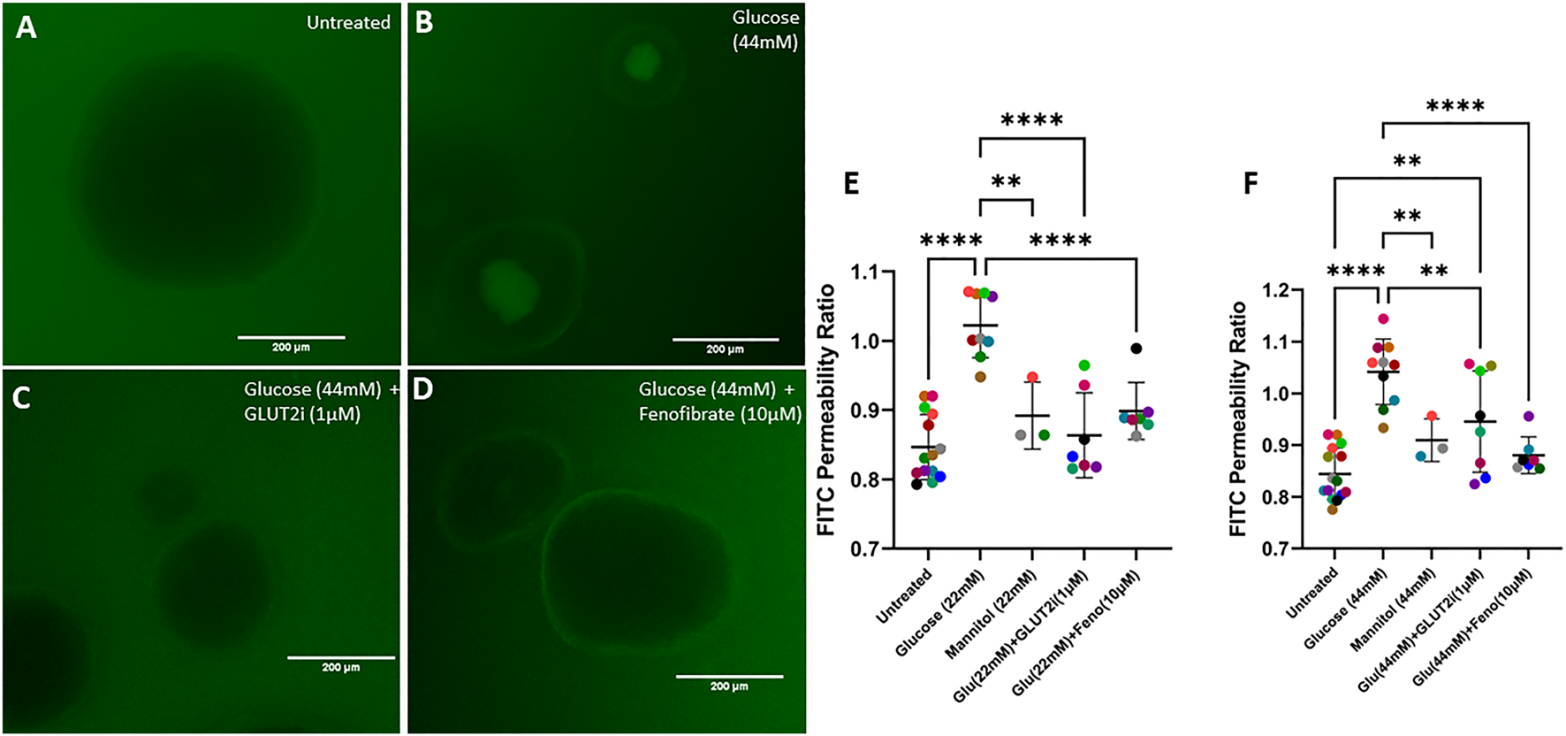
Fenofibrate treatment prevents the increase in barrier permeability that is induced by supraphysiologic glucose concentration. (**A**–**D**) Representative images of feline enteroids following exposure to 4 kDa FITC-Dextran following 24 h treatment with control media (**A**), 44 mM added glucose (**B**), 44 mM added glucose with GLUT2i (**C**), and 44 mM added glucose and fenofibrate (**D**). Images are acquired with a GFP LED light cube (470/525 nm ex/em). Scale bars denote 200 µm. (**E**–**F**) Luminal FITC intensity normalized to basolateral FITC intensity following 24 h treatment with glucose, mannitol, glucose with GLUT2-selective inhibitor, or glucose with fenofibrate. One-way ANOVA (α = 0.05) with Holm-Šídák post hoc analysis determined that treatment with 22 mM or 44 mM additional glucose significantly increased barrier permeability (*p* < 0.0001 for both treatments). Mannitol treatment significantly reduced enteroid permeability compared to matching concentration of glucose treatment for both 22 mM and 44 mM treatements (*p* = 0.0012 and *p* = 0.0097, respectively). Enteroids treated with glucose and 1 µM GLUT2-selective inhibitor displayed significantly reduced barrier permeability compared to those treated with only glucose for both 22 mM and 44 mM treatments (*p* < 0.0001 and *p* = 0.0097, respectively), but co-treatment with GLUT2-selective inhibitor did increase barrier permeability relative to untreated enteroids for 44 mM glucose-treated enteroids (*p* = 0.0034). Enteroids treated with glucose and 10 µM fenofibrate displayed significantly reduced permeability compared to those treated with only glucose for both 22 mM and 44 mM glucose treatments (*p* < 0.0001 for both treatments). Data are presented as mean ± SD with each point representing an experimental mean. Colors represent individual experiments from one organoid line.

We hypothesized that hyperglycemia directly increases the permeability of the feline intestinal epithelium. To test this hypothesis, we modeled hyperglycemia by exposing the basolateral surface of feline enteroids to supraphysiologic glucose concentrations and measured intestinal permeability using a FITC-dextran permeability assay, as previously described^37^. Our data show a significant increase in epithelial permeability when enteroids were exposed to 22 mM and 44 mM glucose above baseline (*p* < 0.0001, *p* < 0.0001, respectively) (Fig. 1). To determine if the effects of hyperglycemia-like conditions on epithelial permeability were driven by increased osmotic pressure, enteroids were treated with equal concentrations of the inert sugar, mannitol^44,45^. Mannitol treatment had no effect on permeability relative to untreated enteroids, though with a smaller sample size of three experiments. Moreover, 22 mM and 44 mM glucose above baseline significantly increased permeability (*p* = 0.0012 and = 0.0097, respectively) compared with its paired mannitol treatment, indicating that increased extracellular osmotic pressure was not responsible for the hyperglycemia-induced increased epithelial permeability.

To determine if increased intracellular concentrations of glucose are causing the increased intestinal epithelial permeability, we utilized a GLUT2-selective inhibitor^41^ (GLUT2i, 1 μM) in conjunction with high glucose concentrations to prevent glucose entry into the epithelial cells. GLUT2i treatment abolished the hyperglycemia-induced rise in intestinal permeability (22 mM glucose, *p* < 0.0001). This suggests that the intracellular increase of glucose concentration is directly driving increased permeability, though this effect was only partially reversed in the 44 mM glucose-treated enteroids (Fig. 1).

We hypothesized that PPARα activation would prevent this hyperglycemia-induced increase in epithelial permeability. When enteroids were exposed to hyperglycemic conditions in conjunction with the PPARα agonist fenofibrate (10 μM), there was no significant increase in permeability relative to untreated enteroids. Treatment with fenofibrate significantly reduced epithelial permeability as compared to supraphysiologic glucose treatment without fenofibrate (*p* < 0.0001 for 22 mM, *p* < 0.0001 for 44 mM).

### Hyperglycemia-like conditions disrupt tight junction morphology, which is prevented by fenofibrate

**Figure 2:**
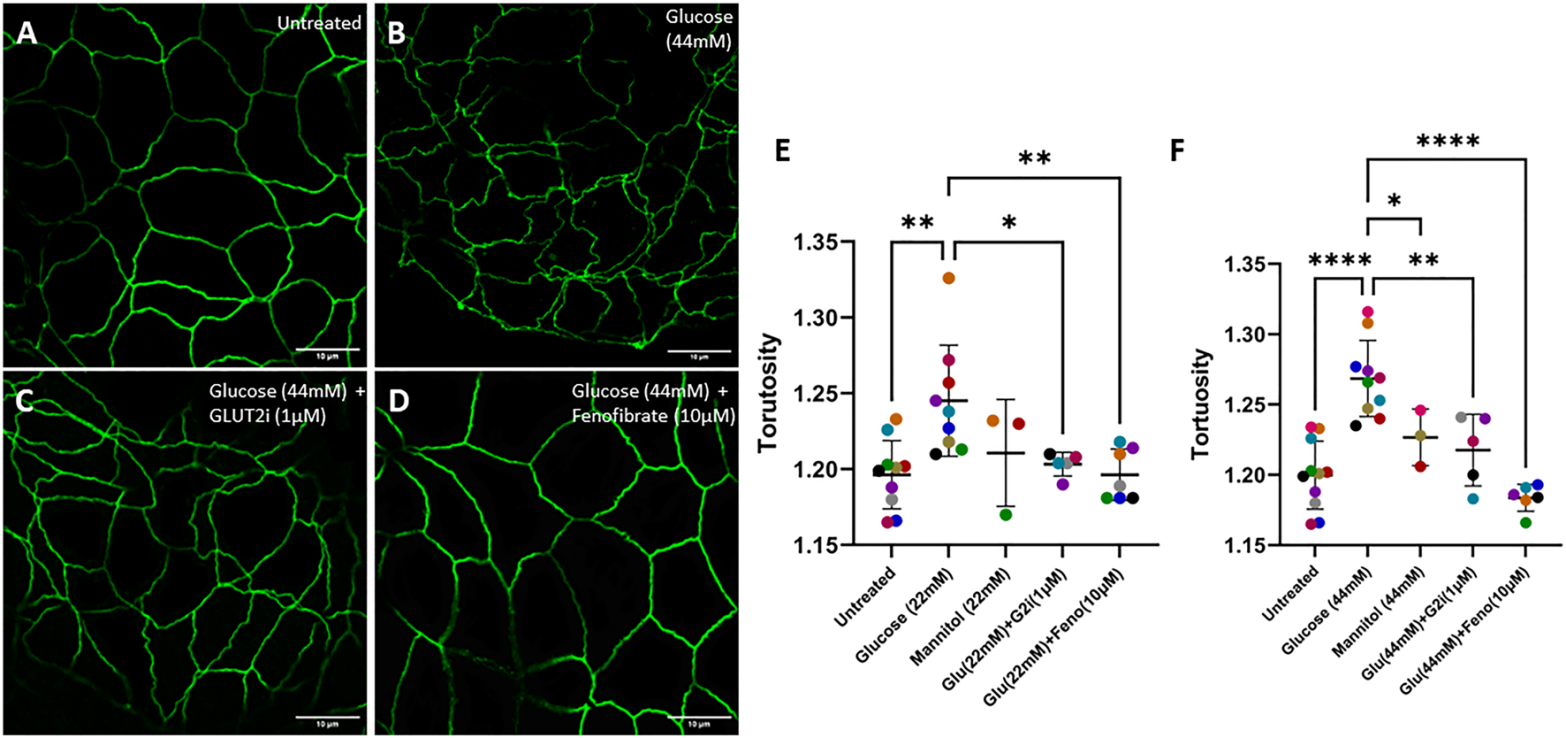
Fenofibrate treatment prevents the increase in tight junction tortuosity that is induced by supraphysiologic glucose concentration. (**A**–**D**) Representative ZO-1 (Alexa Fluor 488) staining of feline enteroids following 24 h treatment with control media (**A**), 44 mM added glucose (**B**), 44 mM added glucose with GLUT2i (**C**), and 44 mM added glucose and fenofibrate (**D**). Scale bars denote 10 µm. (**E**–**F**) Quantification of ZO-1 tortuosity in experimental enteroids. Treatment with 22 mM or 44 mM glucose significantly increased ZO-1 tortuosity (*p* = 0.0023, *p* < 0.0001, respectively) as determined by one-way ANOVA (α = 0.05) and Holm-Šídák post hoc analysis. 44 mM mannitol treatment significantly decreased junctional tortuosity compared to treatment with an equal concentration of glucose (*p* = 0.0416). Enteroids treated with 22 mM or 44 mM glucose and 1 µM GLUT2-selective inhibitor displayed significantly reduced ZO-1 tortuosity compared to those treated with only glucose (*p* = 0.0369 and *p* = 0.0020, respectively). Enteroids treated with 10 µM fenofibrate in addition to 22 mM or 44 mM glucose displayed significantly reduced ZO-1 tortuosity compared to those treated with only glucose (*p* = 0.0053, *p* < 0.0001, respectively). Data are presented as mean ± SD with each point representing an experimental mean. Colors represent individual experiments from one organoid line.

Tight junctions regulate paracellular transport and permeability across the intestinal epithelium. We hypothesized that hyperglycemia increases intestinal permeability by altering tight junction structure. To determine how hyperglycemia influences tight junction structure and spatial conformation we analyzed junctional tortuosity through ZO-1 immunocytochemistry and confocal microscopy image analysis. Hyperglycemia-like conditions significantly increased junctional tortuosity (22 mM and 44 mM, *p* = 0.0023 and *p* < 0.0001, respectively, Fig. 2). When treated with equal concentrations of mannitol, tortuosity was not significantly increased relative to untreated enteroids. While not significant, the mean tortuosity was higher than the baseline, suggesting that there may be a potential effect of increased osmolality, though the role of increased osmolality independent of glucose was not further explored. Moreover, enteroids that were treated with 44 mM mannitol displayed significantly lower tortuosity than those exposed to equal concentration of glucose (*p* = 0.0416), suggesting an effect of glucose independent of overall osmolality. Treatment with GLUT2i reduced tortuosity compared with glucose treatment alone (22 mM and 44 mM, *p* = 0.0369 and *p* = 0.0020, respectively) indicating that increased intracellular glucose concentration directly disrupts tight junction morphology. Treatment with fenofibrate significantly decreased the effect of hyperglycemia on tight junction tortuosity (*p* = 0.0053 for 22 mM, *p* < 0.0001 for 44 mM).

### Hyperglycemia-like conditions reduce cellular proliferation in feline enteroids but has no effect on apoptotic rate

**Figure 3:**
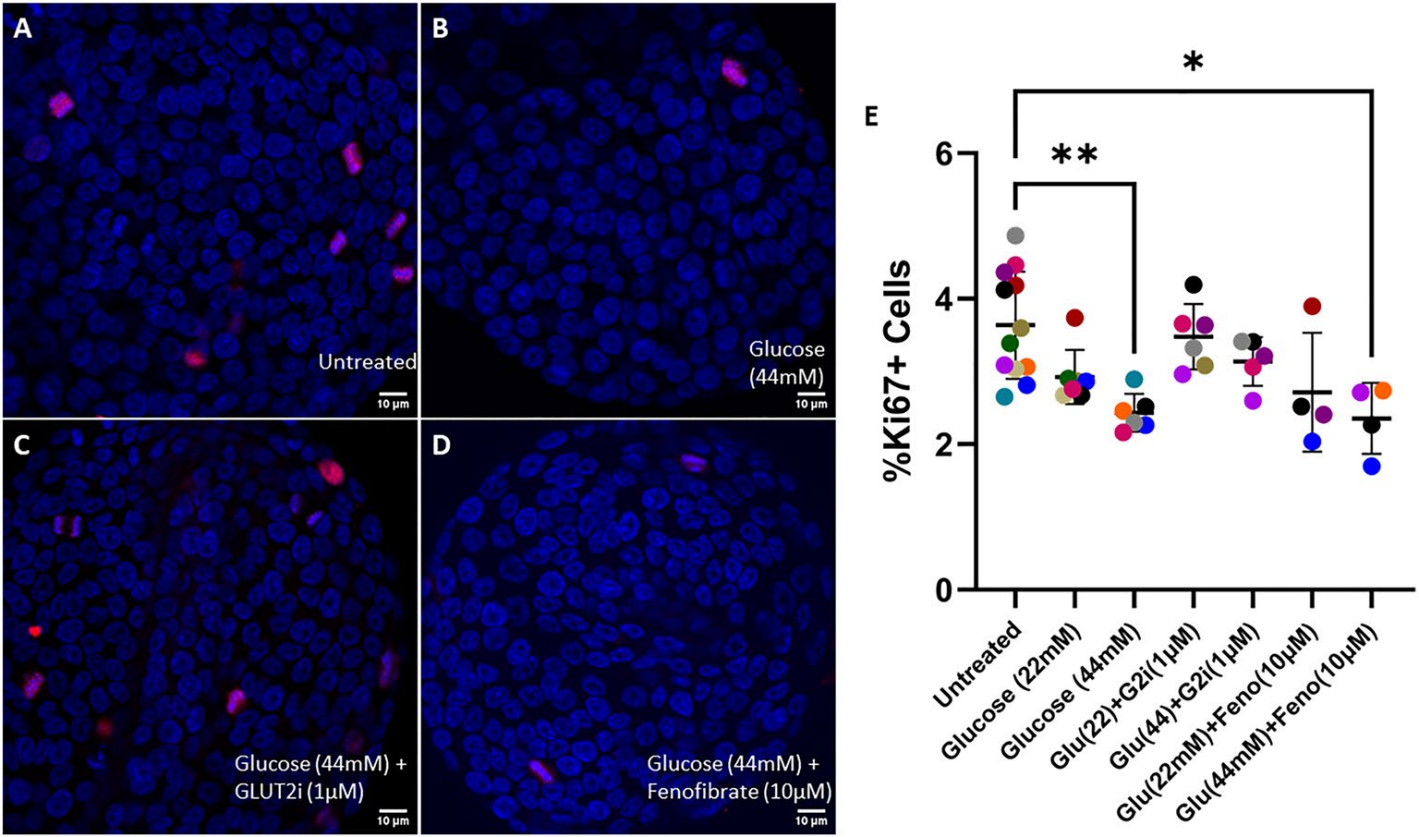
Supraphysiologic glucose concentration reduces cellular proliferation in feline enteroids. (**A**–**D**) Representative confocal images of feline enteroids stained for Ki67 (Alexa Fluor 594) and DAPI following 24 h treatment with control media (**A**), 44 mM added glucose (**B**), 44 mM added glucose with GLUT2i (**C**), and 44 mM added glucose and fenofibrate (**D**). Scale bars denote 10 µm. (**E**) Percentage of proliferating cells measured by confocal microscopy. Treatment with 44 mM glucose with or without fenofibrate induced a significant reduction in proliferation compared to untreated enteroids (*p* = 0.0032 without fenofibrate, *p* = 0.0246 with fenofibrate) as determined by Kruskal–Wallis non-parametric ANOVA (α = 0.05) with Dunn’s post hoc analysis. Data are presented as mean ± SD with each point representing an experimental mean. Colors represent individual experiments from one organoid line.

**Figure 4:**
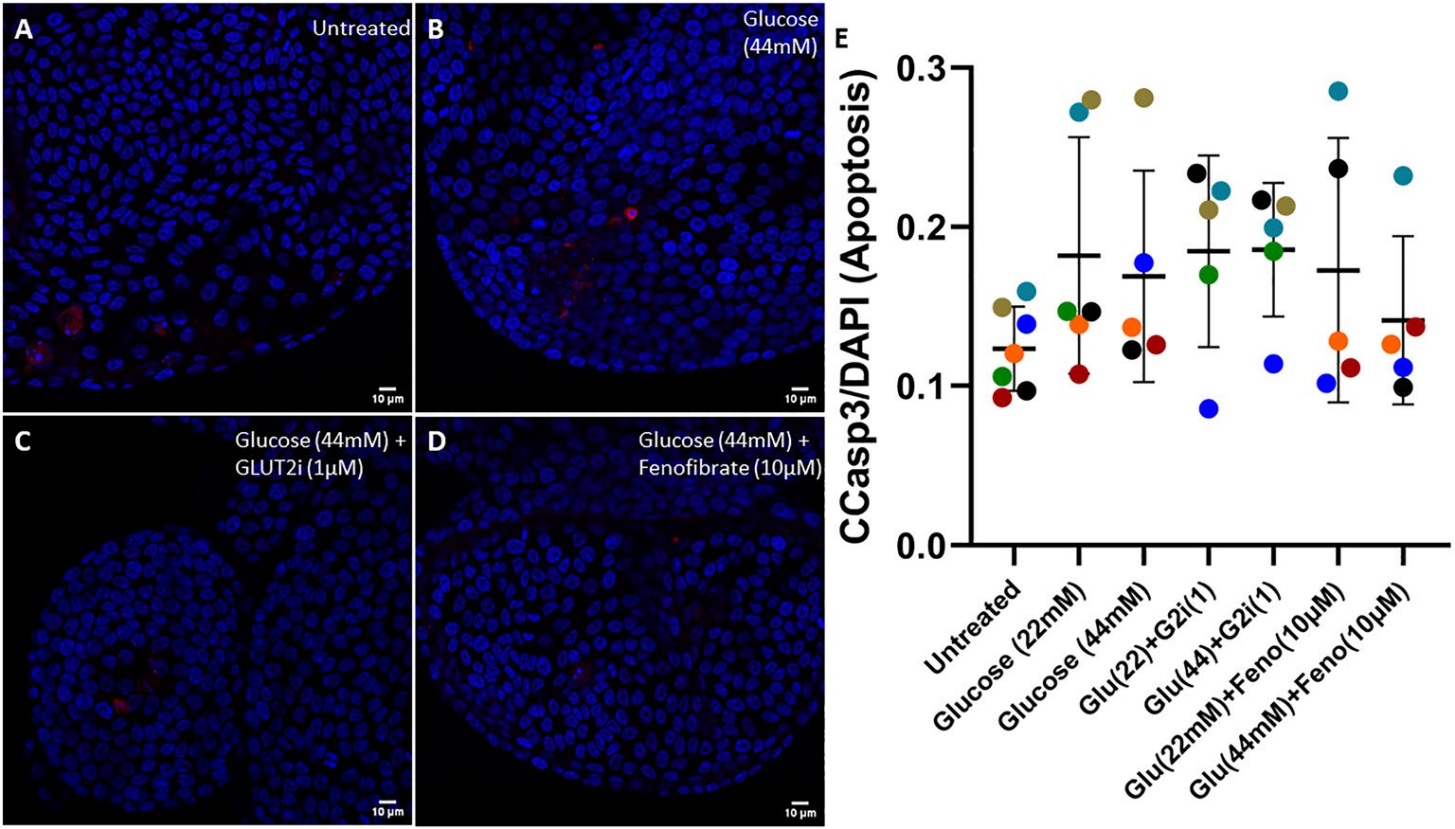
Supraphysiologic glucose concentration does not alter apoptotic rate in feline enteroids. (**A**–**D**) Representative confocal images of feline enteroids stained for Cleaved Caspase-3 (Alexa Fluor 594) and DAPI following 24 h treatment with control media (**A**), 44 mM added glucose (**B**), 44 mM added glucose with GLUT2i (**C**), and added 44 mM glucose and fenofibrate (**D**). Scale bars denote 10 µm. (**E**) Fluorescence intensity of Cleaved Caspase-3 normalized to DAPI measured by confocal microscopy. No significant effect of treatment was detected by Kruskal–Wallis non-parametric ANOVA (α = 0.05). Data are presented as mean ± SD with each point representing an experimental mean. Colors represent individual experiments from one organoid line.

Altered cellular turnover may contribute to epithelial barrier dysfunction. To investigate the effects of hyperglycemia on cellular turnover rate we utilized immunocytochemistry and confocal microscopy to analyze the proportion of Ki67-positive cells (i.e. proliferating cells) and Cleaved Caspase 3 (CCasp3)-positive cells (i.e. apoptotic cells). Treatment with 44 mM glucose above baseline reduced the proportion of Ki67-positive cells relative to untreated enteroids (*p* = 0.0032) (Fig. 3). Treatment with glucose and fenofibrate showed the same effects as treatment with glucose alone. Enteroids treated with glucose (44 mM) and fenofibrate displayed reduced proliferation relative to untreated enteroids (*p* = 0.0246) while there was no significant effect of treatment with glucose (22 mM) and fenofibrate. There was no significant effect of treatment on normalized CCasp3 intensity (Fig. 4).

### Hyperglycemia-like conditions do not alter tight junction gene transcription

**Figure 5:**
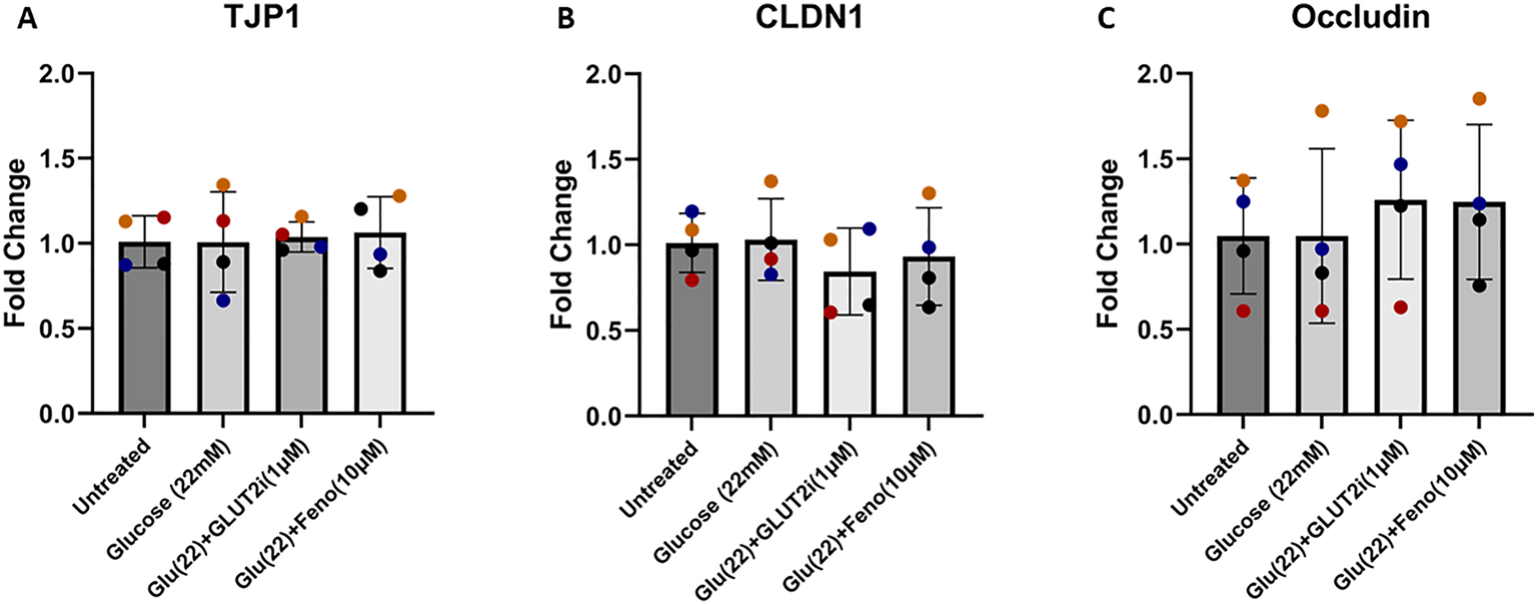
Supraphysiologic glucose concentration, with or without fenofibrate, did not alter expression of tight junction genes. (**A**–**C**) qRT-PCR analysis of the tight junction genes *TJP1* (also known as ZO-1), *CLDN1*, and *OCLN*. No significant treatment effect was observed as determined by one-way ANOVA (α = 0.05). Data are presented as mean ± SD with each point representing mRNA expression normalized to GAPDH. Colors represent individual experiments from one organoid line.

To understand the mechanisms by which hyperglycemia increases intestinal permeability, and how this is improved by fenofibrate, we investigated the effects of hyperglycemic-like conditions with or without fenofibrate on the expression of key tight junction genes via RT-qPCR. Expression of three different tight junction genes were investigated: ZO-1 (*TJP1*), Claudin 1 (*CLDN1*), and Occludin (*OCLN*). However, treatment did not alter tight junction gene expression (Fig. 5).

### Hyperglycemia-like conditions increase PKCα activation, which is prevented by fenofibrate

**Figure 6:**
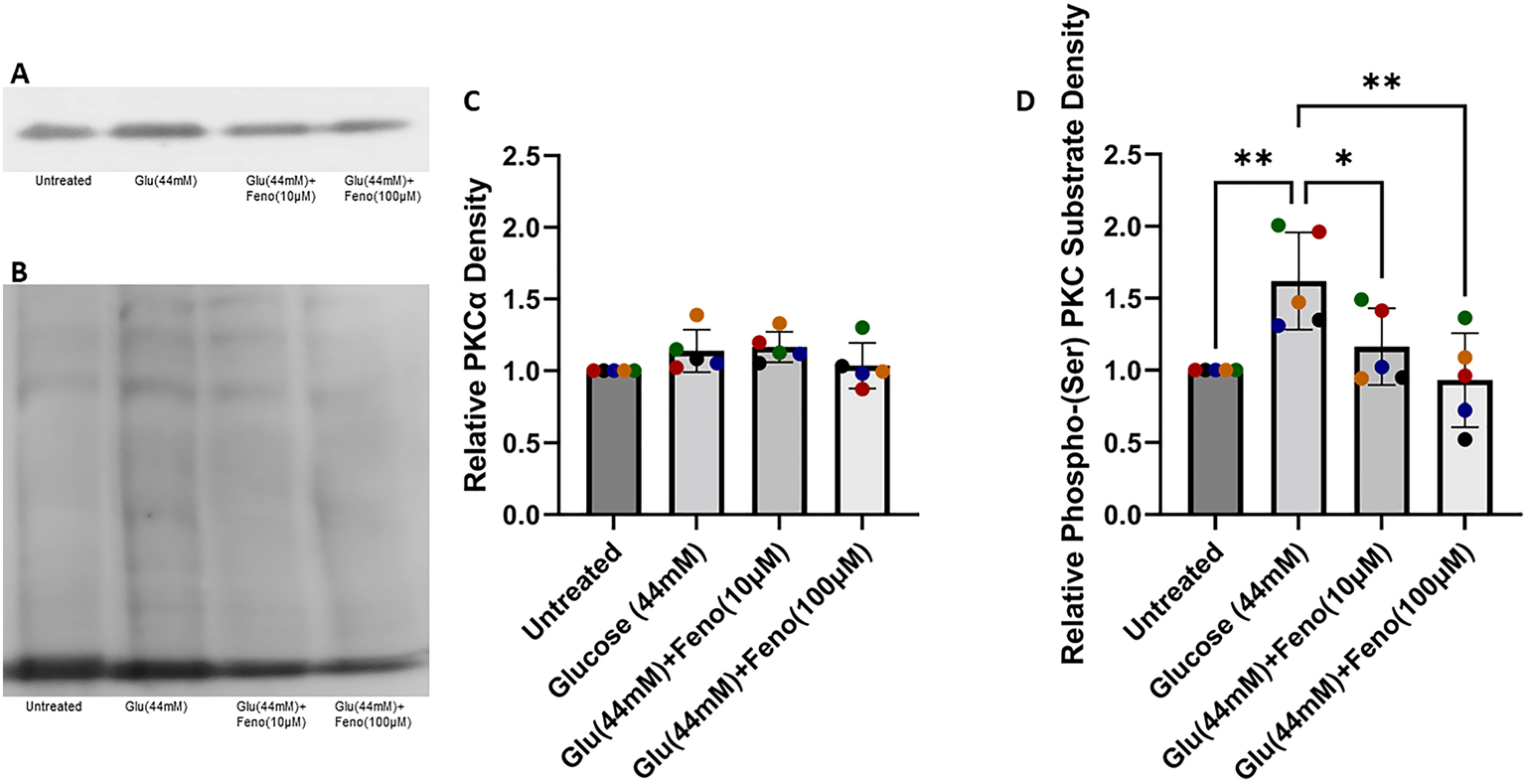
Fenofibrate treatment prevents the increase in PKCα activation that is induced by supraphysiologic glucose concentration. (**A**) Representative western blot probed for PKCα antibody. (**B**) Representative western blot probed for phosphor-(Ser) PKC substrate antibody. (**C**) Densitometry analysis of PKCα. No significant effect of treatment was observed via one-way ANOVA (α = 0.05). (**D**) Densitometry analysis of phospho-(Ser) PKC substrate. Treatment induced a significant effect on phospho-(Ser) PKC substrate expression as determined by one-way ANOVA (α = 0.05) (*p* = 0.042). Holm-Šídák post hoc analysis determined that treatment with glucose (44 mM) significantly increased phospho-(Ser) PKC substrate expression (*p* = 0.0089) elative to untreated enteroids. Treatment with 44 mM glucose and fenofibrate (10 µM or 100 µM) significantly reduced phospho-(Ser) PKC substrate expression (*p* = 0.0492 and *p* = 0.0048, respectively). Lanes include samples derived from the same experiment, processed in parallel. Data are presented as mean ± SD with each point representing protein expression normalized to GAPDH. Colors represent individual experiments from one organoid line.

We did not observe a change in tight junction gene expression; therefore, we investigated an alternative hypothesis for how supraphysiologic glucose concentration and fenofibrate regulate intestinal permeability. Protein Kinase C-α (PKCα) activation is associated with intestinal barrier dysfunction^46,47^, and fenofibrate can inhibit PKCα activation via non-transcriptional pathways^48^. Therefore, we investigated PKCα protein expression and activation in our treated enteroids via western blot analysis. Western blots were performed with a PKCα antibody to investigate the effects of treatment on total PKCα protein levels (Fig. 6A). Additionally, western blots were also performed with a phospho-(Ser) PKC substrate antibody; this antibody binds to PKC substrates with phosphorylated serine residues, thus indicating PKC activity (Fig. 6B). Our data show no significant effect of treatment on overall PKCα protein expression (normalized to GAPDH) (Fig. 6C). However, there was a significant increase in phospho-(Ser) PKC substrate protein expression (all bands within a lane averaged, normalized to GAPDH) for glucose-treated enteroids (44 mM) compared to untreated enteroids (*p* = 0.0089), indicating an increase in PKCα activation without a change in total PKCα (Fig. 6D). When co-treated with 10 μM or 100 µM fenofibrate, phospho-(Ser) expression was significantly reduced compared to glucose-treated enteroids (*p* = 0.0492 and *p* = 0.0048, respectively) (Fig. 6D).

## Discussion

We sought to determine if hyperglycemia directly increases intestinal epithelial permeability, and if any glucose-induced effects may be prevented pharmacologically with the FDA-approved PPARα agonist, fenofibrate. To investigate this, we utilized adult stem cell-derived intestinal organoids (enteroids) to provide an in vitro model that recapitulates native feline intestinal epithelium. We further aimed to determine the effects of hyperglycemia on intestinal epithelial cells independent of inflammation, altered neuronal signaling, or altered smooth muscle motility that may accompany DM. Our model readily enables exposure of the basolateral side of the epithelium to glucose treatment, as is seen during hyperglycemia in vivo, and isolates the effects of glucose on epithelial cells from other confounding effects. With this model, we determined that hyperglycemia-like conditions directly increase intestinal epithelial permeability and increase tight junctional tortuosity while increasing activation of PKCα. We also determined that treating enteroids with fenofibrate prevents glucose-induced changes to permeability and junctional tortuosity, and that fenofibrate attenuates the glucose-induced rise in PKCα activity. This is, to our knowledge, the first study to investigate the independent effects of hyperglycemia on intestinal barrier function in a 3D enteroid model system, elucidate the molecular mechanism of injury, and highlight fenofibrate as a candidate therapeutic agent in this context.

Hyperglycemic-like conditions directly increase the permeability of the intestinal epithelium in feline enteroids as made evident by the increased entry of FITC-dextran (Fig. 1). This is consistent with previous findings of FITC-dextran flux in diabetes-induced mice^8^, though our data isolates the intestinal epithelium from other intestinal cellular components such as immune cells and nervous tissue. Additionally, it has been shown that apical exposure of the human colorectal adenocarcinoma cell line, CACO-2, to high glucose concentrations increases epithelial permeability^49^, which is further corroborated by our findings in feline enteroids which more closely represents a diabetic model with native intestinal epithelial cellular diversity and exposure to hyperglycemia-like conditions via the basolateral pole. We confirmed that increased extracellular osmotic pressure was not the primary cause of the hyperglycemia-induced increased epithelial permeability by incubating our enteroids with equivalent concentrations of mannitol, an inert sugar. The GLUT2 transporter is expressed on the basolateral surface of intestinal epithelial cells and is the primary mode of glucose transport across the basolateral membrane into the circulation. Additionally, it has been shown to facilitate retrograde entry of glucose into intestinal epithelial cells during hyperglycemic conditions^8,50^. We employed a GLUT2-selective inhibitor (GLUT2i) to determine if increased intracellular concentration of glucose is the cause of the observed changes in intestinal epithelial cells^41^. We found that GLUT2i treatment significantly reduces permeability compared to enteroids treated with glucose without inhibition (Fig. 1). However, this did not fully prevent a rise in permeability when inhibited in conjunction with very high (44 mM) concentrations of glucose, as glucose concentrations may have been too high for the effect to be fully counteracted (Fig. 1). The reduced permeability in enteroids treated with mannitol or glucose with GLUT2i compared with our glucose-treated enteroids suggests that increased intra-epithelial concentration of glucose is required for the direct effects of glucose on epithelial permeability.

Tight junctions are a critical component of epithelial barrier regulation and therefore, we aimed to investigate tight junction morphology and gene transcription to understand potential causative mechanisms driving the hyperglycemia-induced increase in epithelial permeability. Previous studies have shown that supraphysiologic concentration of glucose can directly alter the morphological integrity of tight junctions in epithelial cell lines as measured by tortuosity^8,23^, and that increased tight junction tortuosity is associated with increased FITC-dextran permeability in bovine enteroids^37^. Our study shows that hyperglycemia-like conditions increase tight junctional tortuosity, and that this rise is absent when treated with equivalent concentrations of mannitol or co-treated with GLUT2i (Fig. 2). Our study corroborates the effects of glucose on tight junction morphology in other models^8,23^ and further implies the necessity of glucose entry into the epithelial cells as shown by our GLUT2i treatment condition. We also investigated the effects of hyperglycemia on gene expression of tight junction proteins ZO-1 (*TJP1*), Claudin 1 (*CLDN1*), and Occludin (*OCLN*). Claudin 1 immunoreactivity has been shown to increase in diabetic dogs following fenofibrate treatment^23^. High glucose diets in mice increase FITC-dextran permeability while decreasing levels of ZO-1 and Occludin, but also increase levels of inflammatory cytokines, making it difficult to parse the direct effect of high intracellular glucose concentrations, specifically, versus secondary inflammation^22^. As such we investigated the transcription of these three genes, though we found that in the feline enteroid hyperglycemia model there was not a significant change in the transcription rate of tight junction genes. This suggests that the mechanisms driving the observed changes to junctional morphology and epithelial permeability are independent of tight junction gene transcription, though further research must be conducted to determine the potential effects on protein expression in our model.

Because inflammation-induced intestinal barrier dysfunction is associated with lowered cellular proliferation and increased apoptosis^37^, we sought to determine if the effects of glucose on barrier permeability are related to changes in cell turnover in feline enteroids. We found that 44 mM glucose does reduce cellular proliferation. However, there was not a significant reduction in cell proliferation when enteroids were treated with 22 mM glucose above baseline, despite an observed increase in epithelial permeability and tight junctional tortuosity (Fig. 1, Fig. 2). While it has been shown that 72 h exposure to glucose (25 mM) does impair cell proliferation mouse enteroids^51^, we did not observe this effect for our 24 h treatment of 22 mM glucose despite observing a rise in barrier permeability for those treatment parameters. It is likely that the treatment time of our study for 22 mM is too short to observe these effects. Additionally, the apoptotic rate in feline enteroids was not significantly changed due to hyperglycemia-like conditions. Thus, we determine that reduced cellular proliferation may play some role in glucose-induced barrier dysfunction but is not likely the primary causative mechanism driving the effects observed in our study.

In addition to determining the direct effects of glucose on intestinal epithelial permeability, we also sought to determine if hyperglycemia-induced barrier dysfunction could be prevented with the PPARα agonist, fenofibrate, thus aiming to directly address the potential use of fenofibrate as a therapy for DM-related intestinal barrier dysfunction in feline veterinary patients while additionally supporting further investigation into the relationship between fenofibrate, diabetes, and intestinal barrier dysfunction in human patients. Fenofibrate is an FDA-approved drug that is commonly used as a therapeutic to treat hyperlipidemia in human and veterinary patients^52,53^. Moreover, fenofibrate treatment abolished the increase in junctional tortuosity in an induced-DM dog model^23^. PPARα knockout mice display exaggerated barrier dysfunction in response to induced colitis or stress^54,55^. However, because PPARα activation reduces inflammation induced by innate immune cells^56^, it is not determined if PPARα activation by fenofibrate can protect against barrier dysfunction induced by glucose, specifically. We found that fenofibrate does diminish the glucose-induced increase in epithelial permeability (Fig. 1) and tight junction tortuosity (Fig. 2) in feline enteroids observed in our study. These findings corroborate previous work in other models^23,54,55^. Work done in high-fat diet mice has shown fenofibrate to increase expression of tight junction genes^23,57^, which was not observed in our study (Fig. 5). While PPARα is a nuclear receptor that primarily functions as a transcription factor upon ligand binding and heterodimerization with retinoid X receptors^30^, it can additionally directly suppress the activation of Protein Kinase C-α (PKCα)^58^.

PKCα is a serine/threonine kinase that, upon activation, is translocated from the cytosol to the cell membrane and phosphorylates substrates^59^. Excessive activation of PKCα is associated with increased intestinal epithelial permeability mediated by cytoskeletal remodeling resulting in altered cell membrane shape and tight junction leakiness^46,47,60^. In platelets, fenofibrate reduced PKCα activation in the absence of genomic DNA^48^. Furthermore, glucose is known to increase PKC activation through the elevated de novo synthesis of the PKC activator, diacylglycerol, caused by a buildup of glycerol-3-p resulting from increased glucose metabolism^61,62^. Additionally, PKCα is known to alter the structure of the cell membrane, resulting in elevated tight junction permeability, rather than altering tight junction protein gene expression. Our study shows that in feline enteroids, total PKCα protein is not changed, but PKCα activation is elevated as a result of hyperglycemia-like conditions (Fig. 6). Treatment with fenofibrate in conjunction with glucose prevented the hyperglycemia-induced rise in PKCα activation (Fig. 6). This would imply that one causal mechanism for hyperglycemia-induced barrier dysfunction is enhanced PKCα activity. Finally, our study corroborates with previous work showing that fenofibrate inhibits PKCα activation without altering total PKCα protein levels^48^.

Our study is not without limitations. Protocols for feline enteroid generation and culture, as well some of the reagents used in this study are not optimal and as widely validated as similar protocols in humans and standard laboratory animals such as mice. Specifically, our organoids presented a spherical architecture, and the differentiation status of the cells in these organoids was not directly investigated, and as such may influence the results of our experiments compared to organoids with budding structures or differing levels of cellular differentiation. Nevertheless, we believe that our model system has significant advantages over other in vitro model systems such as immortalized human cells lines and feline gut tissue explants. Though our treatments are consistent with other in vitro studies^8,23^, we utilize supraphysiological concentrations of glucose for 24 h, while DM induces chronic hyperglycemia, and often with more subtle blood glucose concentrations with 10-16 mM as a diagnostic range for blood glucose concentration^63,64^. Therefore, further investigation in a chronic, low-grade hyperglycemia, in vitro model is warranted. Additionally, we did not investigate the effects of GLUT2i or fenofibrate in the absence of additional glucose treatment, nor did we investigate the effects of overall changes to media osmolality, independent of glucose, or the effects of our experimental treatments on the size or volume of feline enteroids. Furthermore, we only investigated the effects of treatment on the epithelial cells in our enteroid model, while other components of the intestinal barrier such as the mucus layer or lamina propria cells may also be influenced by hyperglycemia, contributing to overall intestinal barrier dysfunction. We did not quantify the total amount of tight junction proteins. Given our observation that hyperglycemia-like conditions increase intestinal permeability by altering tight junction structure and that fenofibrate treatment mitigates this negative effect, we hypothesized that this effect is mediated by gene transcription regulation. We were surprised to find that this was not the case, and that regulation of tight junction morphology was not transcriptionally regulated in our experimental setting. We identified PKCα activation as a candidate mechanism by which glucose exerts its negative effect on barrier function, and that PKCα is regulated by fenofibrate. We do not currently know if there are changes in translation, protein shuttling, or other potential mechanisms to influence protein distribution or quantity, and we find potential in researching this in our future work.

In conclusion, these data show that hyperglycemia and the downstream increase of intracellular concentrations of glucose directly disrupts the epithelial barrier, alters tight junction morphology, and increases PKCα activation in feline intestinal organoids. Additionally, these effects are diminished by the PPARα agonist, fenofibrate, in a direct and non-transcriptional fashion. These findings show that hyperglycemia, independent of systemic inflammation or altered neural signaling, may contribute to intestinal barrier dysfunction associated with DM in cats and further provides rationale for future veterinary clinical trials to determine the therapeutic benefit of the FDA-approved drug, fenofibrate, in this context.

### Supplementary Information

Supplementary Information 1.

Supplementary Information 2.

## Data Availability

All raw data used to generate figures are provided as source data with this paper. All images generated for this study are available from the corresponding author upon request.
