# Supplementary material for: Fenofibrate reduces glucose-induced barrier dysfunction in feline enteroids
Source: Sci Rep. 2023 Dec 18;13:22558. doi: 10.1038/s41598-023-49874-9 (PMC10728136; doi:10.1038/s41598-023-49874-9)
Supplement: Supplementary file 1 — Supplementary Information 1. [file 41598_2023_49874_MOESM1_ESM.pdf]

Fenofibrate reduces glucose-induced barrier dysfunction in feline enteroids

Charles K. Crawford[1], Aeelin Beltran[1], Diego Castillo[1], Muhammad S. Matloob[1], Mimoli E. Uehara[1], Mary L. Quilici[1], Veronica Lopez Cervantes[1], Amir Kol[1]

[1]University of California, Davis, Department of Pathology, Microbiology, & Immunology, School of Veterinary Medicine, Davis, CA, United States
[*]Corresponding Author: akol@ucdavis.edu

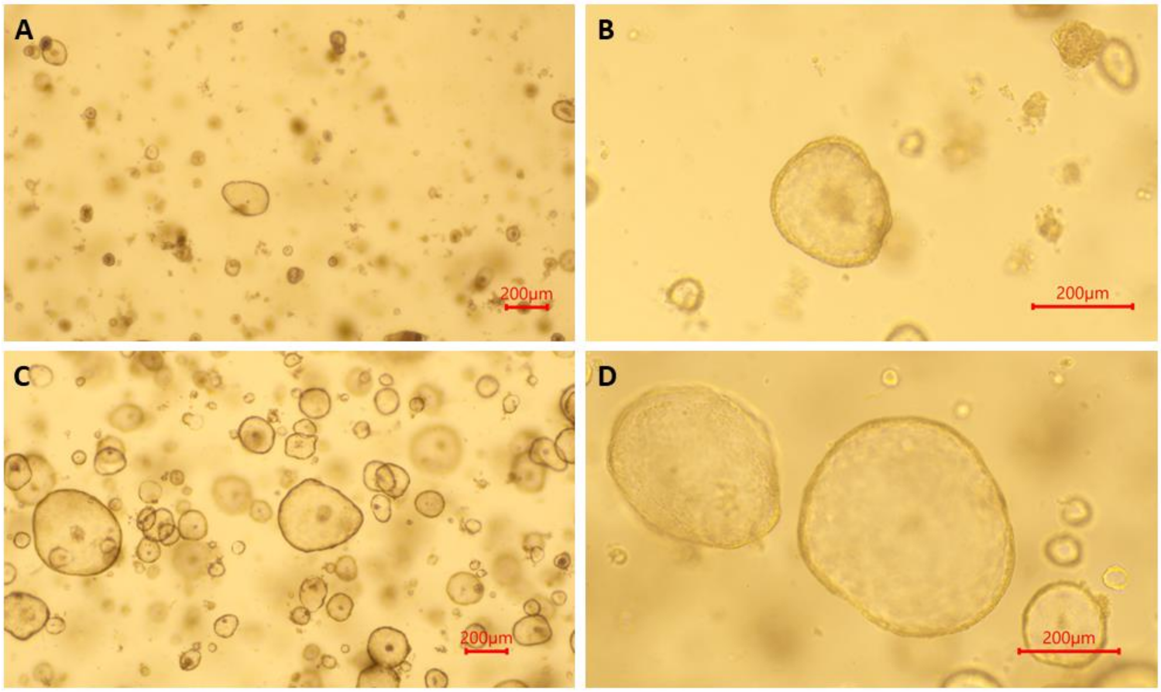

Figure S1. Feline intestinal organoids (enteroids). A) Brightfield image of enteroids 2 days post-passage taken with a 4x objective lens. B) Brightfield image of enteroids 2 days post-passage taken with a 10x objective lens. C) Brightfield image of enteroids 5 days post-passage taken with a 4x objective lens. D) Brightfield image of enteroids 5 days post-passage taken with a 10x objective lens. Scale bars denote 200µm. All images show enteroids in passage-19.

Table S1. Primers used for RT-qPCR

Gene	Forward Primer (5'-3')	Reverse Primer (5'-3')
ZO-1 (TJP1)	CAAGGTCTGCCGAGACAACA	TGCCAGGTTTTAGGATCACCG
Claudin-1 (CLDN1)	TGTCATTGGGGGTGTGACAT	AGCCAGTGAAGAGAGCCTGA
Occludin (OCLN)	CCGCGCTTGGTGTAACAGAT	TCGAACGTGCATGTCTCCAC
GAPDH	AAATTCCACGGCACAGTCAAG	TGATGGGCTTTCCATTGATGA
