# Supplementary material for: Fenofibrate reduces glucose-induced barrier dysfunction in feline enteroids
Source: Sci Rep. 2023 Dec 18;13:22558. doi: 10.1038/s41598-023-49874-9 (PMC10728136; doi:10.1038/s41598-023-49874-9)
Supplement: Supplementary file 2 — Supplementary Information 2. [file 41598_2023_49874_MOESM2_ESM.pdf]

Phospho-(Ser) PKC Substrate

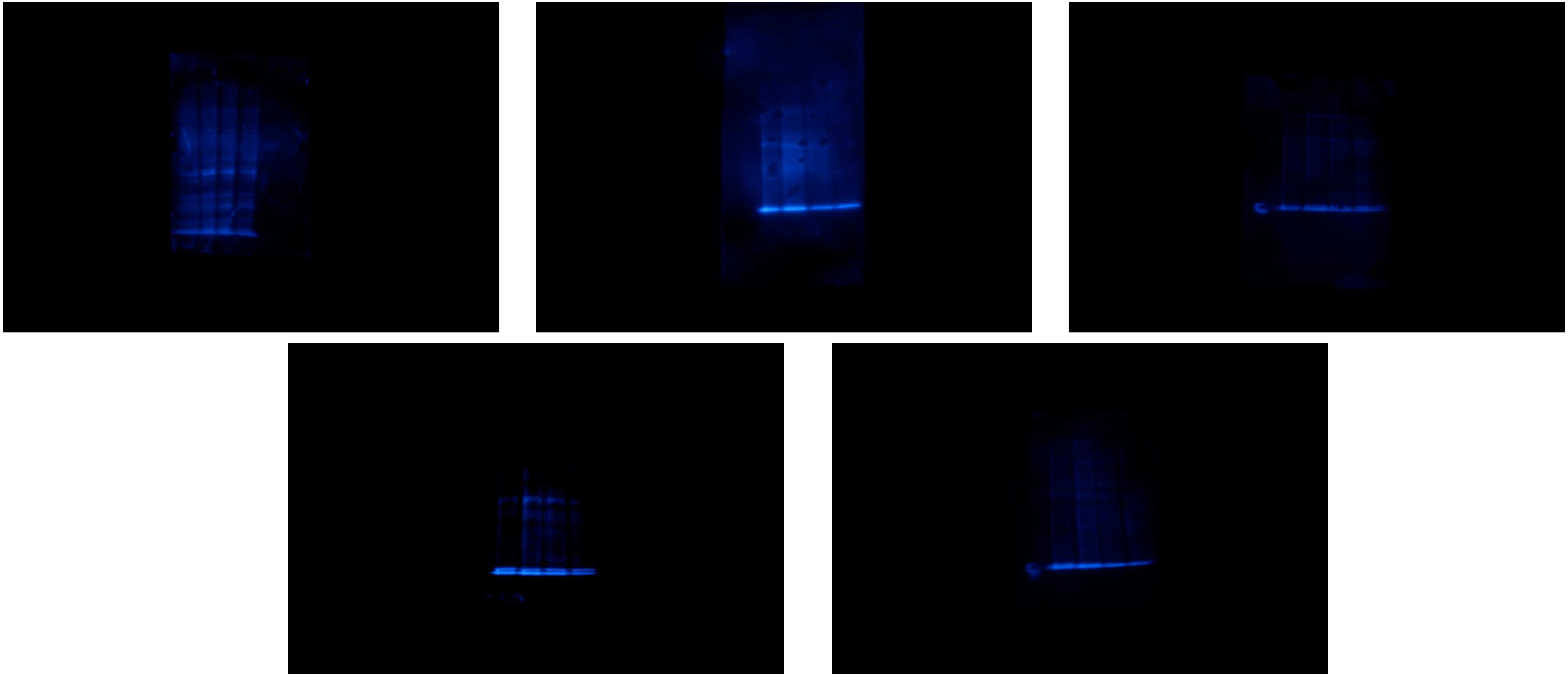

PKCα

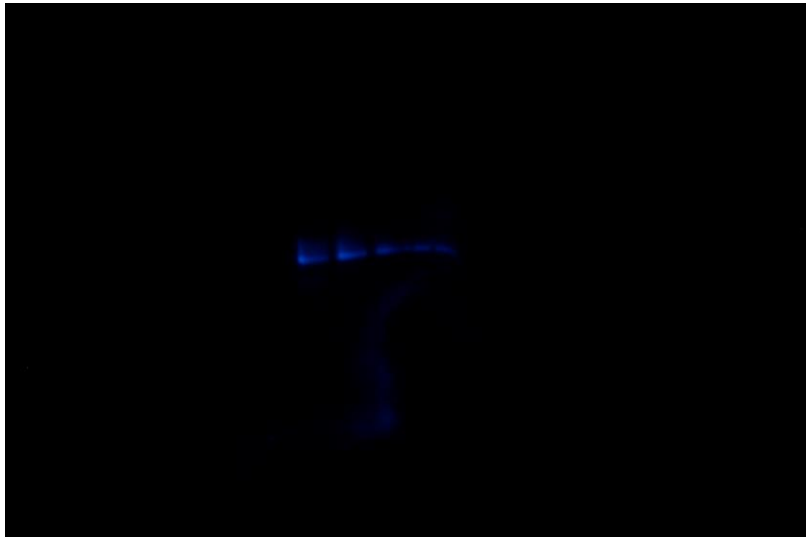

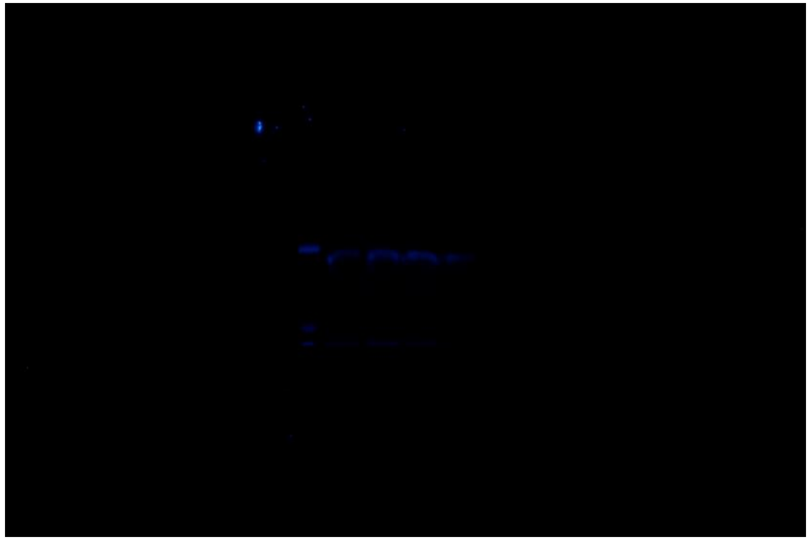

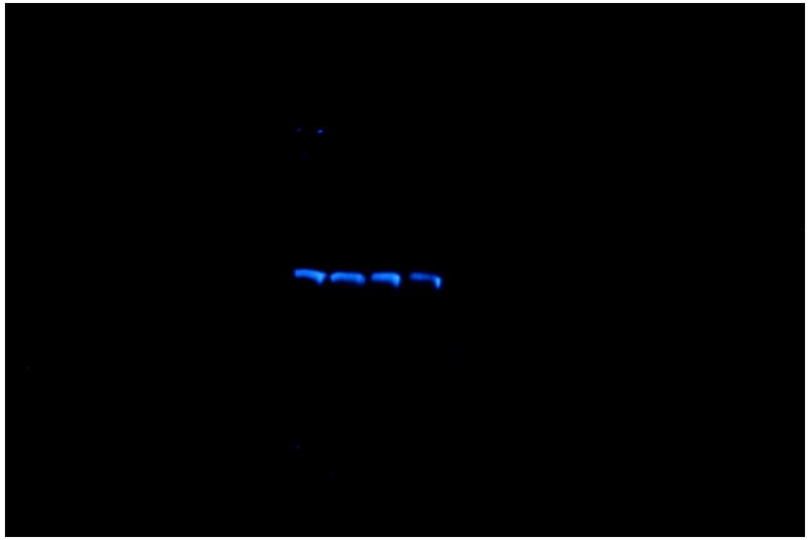

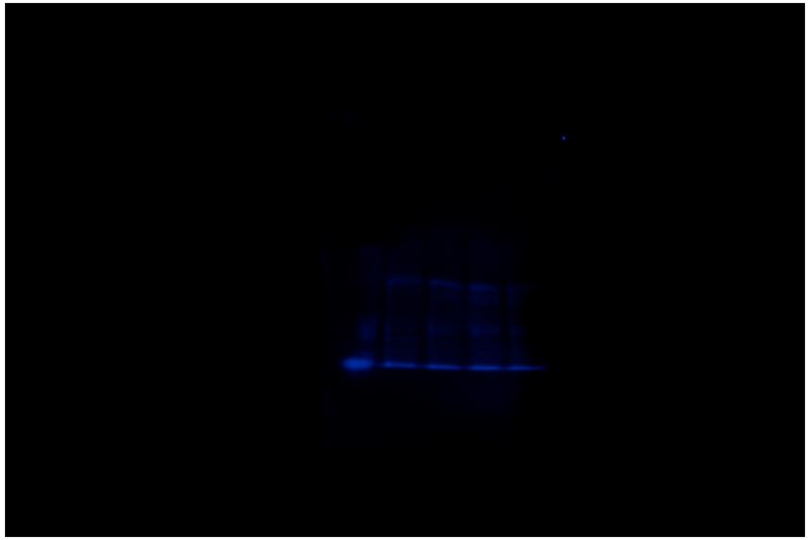

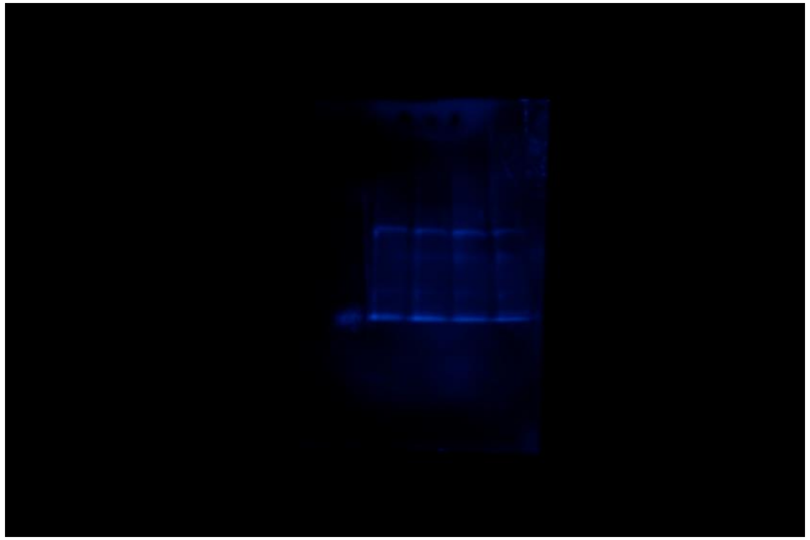

Blots 4 and 5 show both PKCα and GAPDH

GAPDH

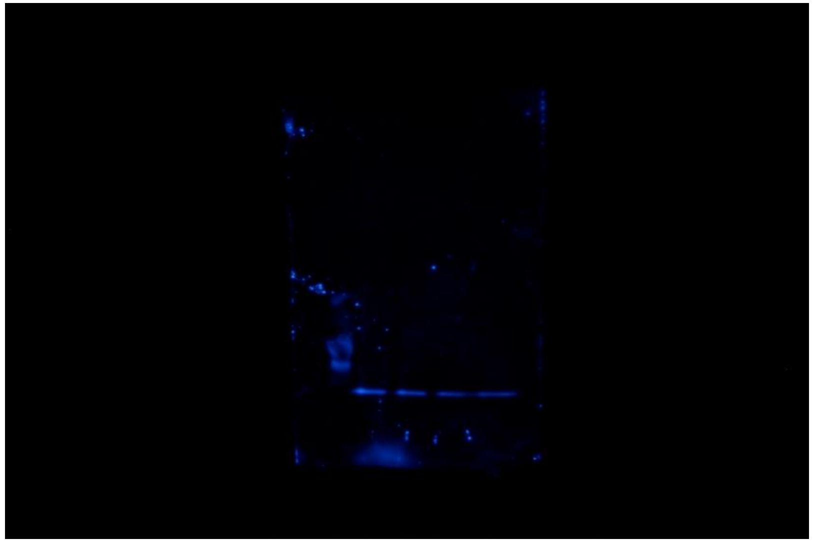
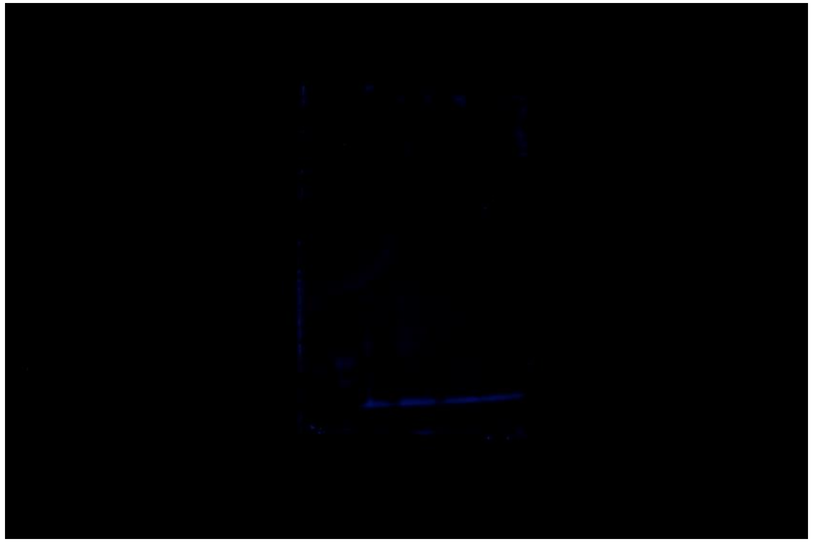
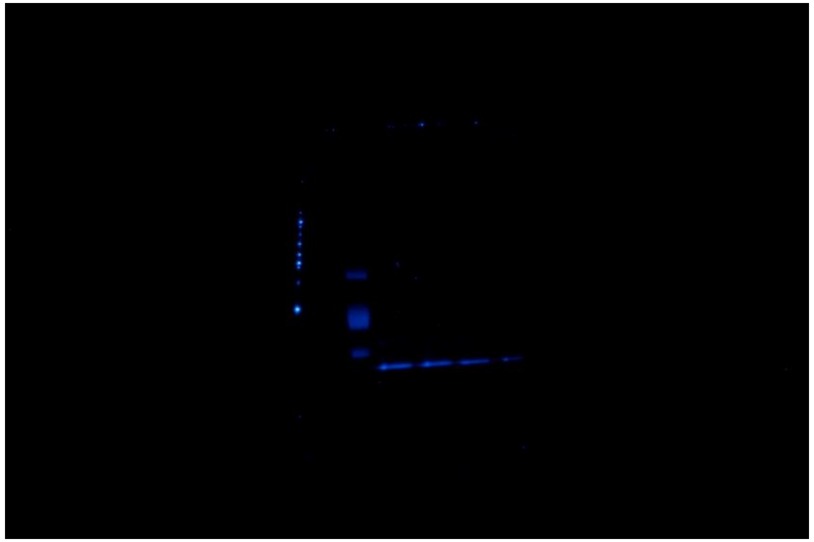
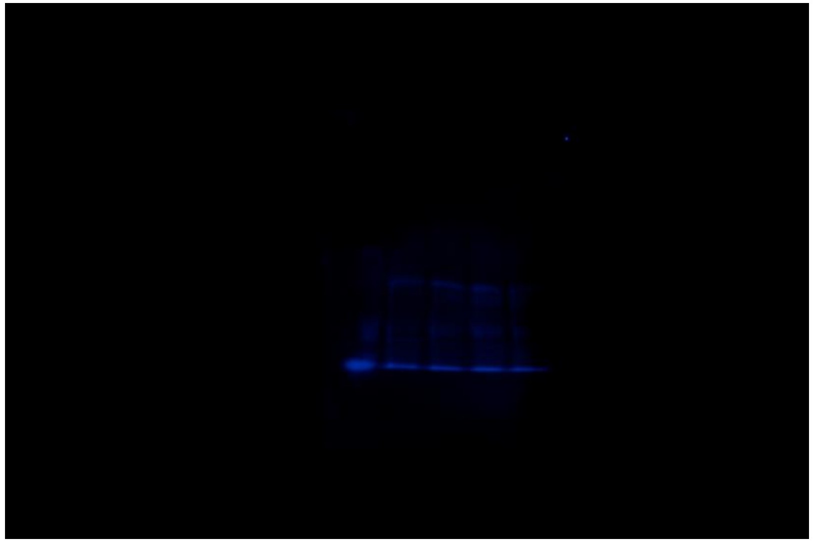
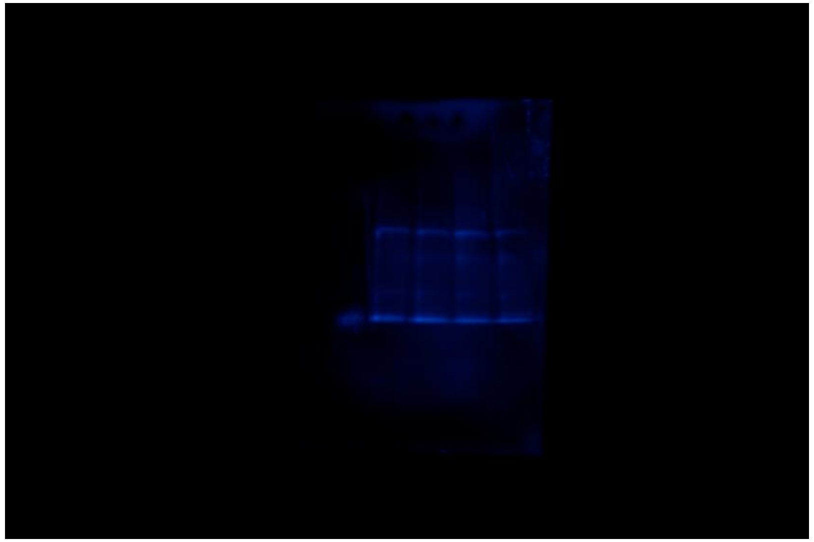

Blots 4 and 5 show both PKCα and GAPDH
